# Targeting Protein–Protein Interfaces with Peptides: The Contribution of Chemical Combinatorial Peptide Library Approaches

**DOI:** 10.3390/ijms24097842

**Published:** 2023-04-25

**Authors:** Alessandra Monti, Luigi Vitagliano, Andrea Caporale, Menotti Ruvo, Nunzianna Doti

**Affiliations:** 1 Institute of Biostructures and Bioimaging (IBB), National Research Council (CNR), 80131 Napoli, Italy; alessandra.monti@ibb.cnr.it (A.M.); luigi.vitagliano@unina.it (L.V.); menotti.ruvo@unina.it (M.R.); 2 Institute of Crystallography (IC), National Research Council (CNR), Strada Statale 14 km 163.5, Basovizza, 34149 Triese, Italy; andrea.caporale@cnr.it

**Keywords:** protein–protein interaction, synthetic combinatorial approaches, peptides, peptidomimetics

## Abstract

Protein–protein interfaces play fundamental roles in the molecular mechanisms underlying pathophysiological pathways and are important targets for the design of compounds of therapeutic interest. However, the identification of binding sites on protein surfaces and the development of modulators of protein–protein interactions still represent a major challenge due to their highly dynamic and extensive interfacial areas. Over the years, multiple strategies including structural, computational, and combinatorial approaches have been developed to characterize PPI and to date, several successful examples of small molecules, antibodies, peptides, and aptamers able to modulate these interfaces have been determined. Notably, peptides are a particularly useful tool for inhibiting PPIs due to their exquisite potency, specificity, and selectivity. Here, after an overview of PPIs and of the commonly used approaches to identify and characterize them, we describe and evaluate the impact of chemical peptide libraries in medicinal chemistry with a special focus on the results achieved through recent applications of this methodology. Finally, we also discuss the role that this methodology can have in the framework of the opportunities, and challenges that the application of new predictive approaches based on artificial intelligence is generating in structural biology.

## 1. Introduction

Protein–protein interactions (PPIs) regulate a complex and intricate number of cellular processes and signaling pathways, including signal transduction and cellular metabolism. PPI may be roughly divided into two different groups depending on the role played by the protein partners and the timescale of their association. The first class includes partnerships underpinned by very strong inter-molecular interactions, such as those occurring in macromolecular complexes, while the other includes the transient associations that mediate signaling pathways and regulatory processes as well as immune suppression, viral infection, and replication [1,2,3]. A further classification of PPI may be based on the rigidity or flexibility of the interacting partners. Indeed, in addition to PPIs involving rigid and well-structured “static” proteins, there is increasing evidence that functional PPI may also involve partners endowed with extremely dynamic attitudes [4]. Indeed, in recent decades, the widely accepted structure–function paradigm [5], in which the folding in precise three-dimensional (3D) structures is a fundamental pre-requisite for protein functionality, has been re-modulated to include the large class of proteins playing key functional roles without adopting, either globally or locally, well-defined structures [6]. These proteins, known as intrinsically disordered proteins (IDPs), or their intrinsically disordered regions (IDRs), are highly dynamic and often undergo conformational change following ligand binding [7,8]. This peculiar behavior allows a single protein to interact with different biological targets, frequently with high specificity and selectivity [9]. The presence of dynamic partners in PPI has other advantages in signaling networks. Conformational fluctuations occurring in dynamic interaction motifs can foster post-translational modifications and/or interactions with other target proteins. A remarkable example of the interplay between static and dynamic PPI is the mechanism p120 catenin uses to regulate the stability of cell–cell adhesion. This mechanism relies on the binding of the intrinsically disordered cytoplasmic tail of cadherin through both “static” and “dynamic” interfaces [10]. The central amino acid sequence of the cadherin cytoplasmic tail strongly binds p120 through a well-structured “static” interface. On the other hand, the unfolded region of the cadherin tail flanking the protein N-terminus also weakly but dynamically interacts with p120. Fluctuations of this second “dynamic” binding site provide a marginal contribution to the binding affinity, but play a crucial function in determining cadherin cell fate by masking a Leucine–Leucine motif which hinders internalization via cadherin-mediated endocytosis [10]. Given the fundamental role played by protein partnerships in cells, networks of PPI are tightly regulated to deliver specific signaling over time and space. Not surprisingly, perturbing PPIs or changing their cellular abundance and distribution may promote diseases, such as cancer, infections, and neurodegenerative diseases [11,12,13,14,15,16,17,18]. Given the paramount relevance of PPIs in both physiology and pathology, their modulation has enormous potential not only in basic research to study biological events at the molecular level, but also in developing new therapies where PPI modulators are attractive molecules for therapeutic interventions. For their physicochemical and functional features, which include high selectivity and reduced immunogenicity, peptides represent ideal candidates or at least precursors to develop effective modulators of PPIs. There is a considerable amount of scientific literature evidencing the ability of peptides to modulate cellular processes regulated by PPIs and supporting the idea that they can become valuable therapeutic assets [19,20,21,22,23,24,25]. With the ever-increasing need of discovering peptide-based PPIs modulators, several approaches are being developed. Among the others, chemical peptide library approaches are attracting the interest of the scientific community.

The present review aims at providing an overview of the contribution provided over the years using synthetic combinatorial peptide libraries to advance both the understanding of basic physio-pathological pathways and the drugs’ discovery. After a global description of PPIs and of the tools commonly used to identify and characterize them (Section 2), we describe in detail the advantages and the drawbacks of using peptide-based molecules in modulating protein–protein partnerships compared to other classes of compounds (Section 3 and Section 4). An overview of the experimental combinatorial strategies used to identify and develop peptides as PPI modulators is provided in Section 5. In Section 6, some recent applications of combinatorial peptide chemistry approaches for generating peptide-based compounds that target PPIs are illustrated. Finally, the impact that these methodologies had and will have on drug development in the future is discussed, also considering the challenges and the opportunities generated by the use of newly implemented machine-learning predictive tools that are revolutionizing structural biology (Section 7).

## 2. Identification of Protein Partnerships and Characterization of PPI

As outlined above, the study of PPIs is of fundamental importance for understanding physio-pathological cellular functionality. However, their reversible and frequently transient nature makes the identification of the proteins involved in these interactions a non-trivial task. Over the years, several methods have been developed to pursue this goal. These approaches can be classified into three distinct types: in vitro, in vivo, and in silico. In vitro techniques are experimental procedures performed in a controlled environment outside a living organism. Among others, tandem affinity purification-mass spectroscopy (TAP-MS) and co-immunoprecipitation (co-Ip) have been used for a long time in the identification of protein partnerships [26,27,28]. TAP-MS is an improved variant of affinity purification mass spectrometry (AP-MS) [29]. As with AP-MS, it allows the isolation of protein complexes under native conditions. The method involves the fusion of the TAP tag, which consists of a calmodulin binding peptide (CBP), a TEV protease cleavage site, and two Protein A domains to the C-terminus of the bait protein. Compared to AP-MS, TAP-MS is characterized by a lower background of contaminating proteins since it implies a two-step isolation process followed by MS analysis [30]. Co-Ip is principally used to validate PPIs, and is based on target protein-specific antibodies immobilized on solid surfaces used to extract the entire protein complex bound to the biological target [26]. Other in vitro techniques have been developed for determining the binding affinity of PPIs. Methods such as Förster resonance energy transfer (FRET), time-resolved (TR)-FRET [31,32,33,34], protein-fragment complementation assays (PCAs) [35], which require labelling of the interacting components, have been extensively used to study PPIs in a cellular context as well [33,35,36,37]. However, covalent labeling may alter the structural properties of the molecules interfering with the proper protein recognition. Furthermore, it is also a time-consuming process. Label-free methods such as surface plasmon resonance (SPR), biolayer interferometry (BLI), quartz crystal microbalance (QCM), and isothermal calorimetry (ITC) are currently used in vitro to overcome these difficulties and are attracting increasing interest [37,38,39,40]. Label-free techniques which exploit well-known physical phenomena have unique advantages, such as low analyte consumption and sample recovery, high sensitivity, small working volumes, high throughput, measurements of various binding parameters and real-time monitoring.

In SPR experiments, a ligand coated on a sensor surface is illuminated with polarized light at a specific angle that excites surface plasmons (SPR angle). Capture of a binding molecule by the ligand induces a change in the SPR reflection angle which gives a direct and real-time measure of the amount of bound material, expressed as response units (RU). Real-time SPR experiments provide both kinetic (kon and koff) and thermodynamic (KD) information by fitting the association and dissociation curves [38].

In BLI experiments, similarly to SPR, the ligand is immobilized on a sensor surface and the binding event is detected using a fully optical method. Additionally, this technique allows the determination of the kinetic rate constants and binding affinities of the molecular interactions. In contrast to SPR, where the binding surface is exposed to a continuous flow, the BLI sensor tip is immersed in vortexed wells containing the analytes at different concentrations [38].

The QCM is a valid alternative to SPR. It is an acoustic sensor based on a piezoelectric crystal, consisting of a thin quartz disk with electrodes plated on both disks connected to an oscillating circuit. Binding of the analyte to the immobilized ligand results in a resonance frequency shift that is recorded over time and generates sensorgrams from which the equilibrium binding constants and affinity constants can be derived [41,42,43,44].

ITC uses stepwise injections of an analyte into a tightly thermostated calorimetric cell containing the ligand. Binding of the analyte generates a reaction heat exchange that is a function of the affinity. Measurements at various concentrations provide a complete thermodynamic characterization, as the binding affinity, and the stoichiometry of reactions. However, in contrast with optical techniques, ITC requires a large quantity of reagent (high micromolar range) and is therefore less amenable to HTS [39].

In vivo techniques are performed on whole living organisms [45]. The main in vivo method for detecting interacting proteins is the classical yeast two-hybrid (Y1H) and its modified versions (Y2H, Y3H) [46]. In this technique, a transcription factor is split into its two domains, the DNA-binding domain (BD) and that involved in catalytic activation (AD). The BD is fused to the target protein, which is used as bait, whereas the AD is joined with a set of selected potential binding partners, called the prey. If bait and prey associate, the transcription factor is reconstituted and activates the transcription of a reporter gene, which generally encodes for a protein causing a measurable color change (e.g., ß-galactosidase).

In addition to the experimental methods, a growing number of computational methods that predict protein association with partners are being proposed [47]. In this field, sequence-based and coevolution-based methods can be used to predict the interaction probability. Insights into residue coevolution derived from multiple sequence alignment of homologs may be exploited to predict protein partners assuming that partnerships usually coevolve to preserve their function [48]. For example, orthologous associations may be inferred assuming that proteins known to interact in one species can also interact in other species where these are conserved (interologs) [49]. This scenario is rapidly evolving since approaches based on machine-learning techniques able to predict with good accuracy protein partnerships based on their sequences and scalable at the genome level are becoming readily available [50].

Once interacting proteins have been identified, the definition of the structural basis of their recognition may be undertaken using both experimental and computational approaches. Traditionally, X-ray diffraction and NMR spectroscopy are the experimental techniques most frequently used to achieve this task. In recent years, impressive technological and methodological advancements have been made to improve the accuracy of structural models obtained via single particle cryogenic electron microscopy (cryo-EM) sufficient for providing an atomic-level view of protein–protein interfaces. Although each of these techniques has critical limitations, they collectively constitute the toolbox to elucidate the structural determinants underpinning the intricate network of partnerships that characterize biological processes.

Computational approaches can also provide important insights into the atomic level characterizations of PPIs. Depending on the type of information exploited, these approaches are classified into two broad classes: sequence- and structure-based methods. Since sequence-based approaches are based only on the primary structure of the interacting partners, their application is universal. In one of the early applications of this methodology [51], which was based on a learning algorithm–support vector machine, the authors were able to predict not only protein–protein partnerships, but also PPI networks. Over the years many other methods, mostly based on machine-learning approaches, have been proposed (see for a recent update of the field “Recent advances in predicting protein–protein interactions with the aid of artificial intelligence algorithms”) [52].

The application of structure-based methodologies has been strongly favored by the recent technological advances recently achieved in structural biology [53]. The huge amount of experimental structural information has been exploited to generate large databases of PPI [54] that constitute a solid base for the further development of structure-based approaches [52]. It is important to note that predictive approaches based on machine learning, which provide reliable three-dimensional models of millions of proteins, have also shown their effectiveness in the prediction of the structure of multi-chain protein complexes [55,56,57,58,59]. These advances, unthinkable just a few years ago, suggest that shortly, we will have detailed atomistic representations of most PPI involved in human physio-pathology together with a wealth of impressive information useful for the structure-based design of new molecules for diagnostic and/or therapeutic applications. In this scenario, structure-based approaches may become as universal as sequence-based methodologies. In addition to these contributions, machine-learning approaches also provide important information on the in silico identification of druggable sites in proteins [60,61], including allosteric ones [62].

Computational approaches have also been largely investigated for the high throughput virtual screening (HTVS) of large collection of compounds. In a recent and comprehensive review, methods more frequently used in the last decade have been surveyed [63]. However, HTVS approaches are beyond the scope of this review; therefore, we suggested to refer to this paper to gain insights on software most commonly used for preparing peptide libraries and for their screening techniques and on the pitfalls accompanying them, which can always be around the corner when no wet validations are performed.

## 3. Molecules Targeting PPI

Although the targeting of PPIs has long been recognized as a promising therapeutic approach for most diseases, the development of compounds that modulate their pathological activity has only taken off in the last 20 years. Indeed, the targeting of PPIs was initially attempted through the screening of small molecule libraries. However, while this approach worked very well for enzyme or receptor-ligand interaction inhibition, it most often did not work in the case of PPIs. Few examples of successful PPI targeting with small molecules are known to date [64]. One well-studied system is the targeting of MDM2-p53 complex. p53, a human transcription factor, plays a crucial role in controlling dysregulated cell growth and suppressing tumor-associated phenomena [65,66,67]. In many human cancers, its tumor-suppressor function is impaired by loss-of-function mutations or due to overexpression of its main negative regulator (MDM2). MDM2 is indeed overexpressed in 40–60% of human sarcomas as well as many other solid and hematologic malignancies, making it a very attractive target for anti-tumor drugs. Many small molecules targeting the MDM2-p53 interface have been proposed and some are currently in clinical trials [68,69]. The development of anti-MDM2-p53 inhibitors has been facilitated by the relatively small binding surface area underpinning the interaction and by the presence of an obvious binding cavity (pdb code: 4HFZ). This is however not generally the situation for most PPIs which are instead characterized by broad and flat interfaces in the range of 1000–2000 Å^2^, considerably larger compared to those of small molecule binding sites covering approximately 300–500 Å^2^ [70]. Moreover, most PPIs lack definite binding pockets and amino acid residues involved in the interaction are not adjacent but are placed in the proper orientation due to the complex 3D organisation of the protein. For all these aspects, PPIs have long been considered “undruggable”, and of 64,000 PPIs identified in the human interactome to date, only very few modulators of PPIs have been approved for clinical uses [18].

Significant results on PPI targeting have been obtained by developing highly specific monoclonal antibodies (mAb) that recognize the interaction region in PPIs. The global mAbs market size has reached about USD 162.47 billion in 2021, and is expected to grow up to USD 390.58 billion by 2030 [71,72]. The use of monoclonal antibodies (mAbs) as modulators of PPIs has several advantages. mAbs have high specificity and high affinity for the target and can recognize specific conformations. However, due to their high molecular weight and difficulty in penetrating cell membranes, they mostly find applications for targeting surface proteins. Developing specific and effective antibodies against membrane proteins may have several limitations, including the availability of the antigen in its active form and in sufficient quantities for immunization and screening, and the need for time-consuming and expensive technologies [73,74]. Additionally, the use of mAbs as therapeutics agents has considerable drawbacks related to production costs and immunogenicity [75,76].

Aptamers, made of chemically prepared single-stranded RNA or DNA oligonucleotides, are also increasingly finding application as PPI modulators [77]. They are also known as chemical antibodies for their ability to fold into complex 3D conformations, which give rise to huge numbers of different structures with different recognition properties. Compared to antibodies, RNA/DNA aptamers are significantly cheaper to produce using routine chemical synthesis and, most importantly, show lower immunogenicity and toxicity compared to mAbs. However, the low chemical and biological stability significantly limits their use as PPI modulators [78].

Developing new and effective agents targeting PPIs thus remains an intriguing challenge still today [79,80,81] and their growing need often remains unmet.

Peptides, for their ability to mimic the protein regions involved in the mutual binding surfaces, represent the ideal molecule’s class for addressing PPIs modulation [82].

## 4. Peptides as Effective PPI Modulators

Enormous progress has been made in the design and development of PPI-targeting agents, predominantly focusing on the interaction’s “hot spots”, i.e., the residues mainly involved in the interaction and conferring most of the binding energy [83,84].

Peptides as tools to modulate PPIs are thus increasingly attracting the interest of the scientific community. If properly structured, they can mimic natural interaction motifs and can cover large interaction areas. In addition, with the flexible side chain groups, they can reach interaction sites that lack deep binding pockets and may work as efficient PPI modulators where the small molecules often fail. Furthermore, compared to antibodies, peptides are easily prepared and modified via cheap and fast synthetic processes and can also modulate the biological functions of intracellular targets difficult to reach with antibodies. Peptides can also best regulate the interactions mediated by IDPs. The conformational plasticity of the disordered regions of proteins is in fact definitely more targetable by highly flexible peptides than by rigid small molecules [9]. However, despite the many advantages of using peptides as PPI modulators, to date, peptides only represent 2% of the pharmaceutical drug market [85]. The major limiting aspect of using peptides in therapy is the high biochemical instability in vivo. To overcome these limitations, peptides are often translated into peptidomimetics to reduce their peptide nature, a process known as “depeptidization” [86,87]. Depeptidization is a crucial step in developing effective peptide-based therapies as it leads to increased plasma half-life and decreased enzymatic metabolism and renal elimination. Peptidomimetics are the best option to overcome the problems of poor bioavailability and of stability toward proteolytic enzymes, increasing the duration of activity. Peptidomimetics are structurally modified peptides, possessing the essential recognition elements (pharmacophore) of a natural precursor, and, as such, produce the same biological effect. Other relevant properties, such as target selectivity or potency, are also often substantially improved by passing to peptidomimetics. For example, the incorporation of D-amino acids and/or unnatural amino acids into biologically active peptides improves their metabolic stability. Similarly, cyclization, including side-chain-to-side-chain, head-to-tail, head-to-side-chain, and side-chain-to-tail cyclization, also improves metabolic stability and conformational properties. An elegant example of side-chain-to-side-chain cyclization is the stapling of linear peptides to mimic functionally important secondary structure motifs, a modification that improves biological activity and metabolic stability [88]. The conjugation of peptides to cell penetrating peptides (CPPs) or to fatty acids, instead, increases cellular uptake and prolongs their half-life [89,90].

To date, more than 60 peptidomimetic drugs have received regulatory approval from the FDA as therapeutics for different diseases, including metabolic, cardiovascular diseases, and cancer. Additionally, more than 170 peptides are in active clinical trials, and in many others preclinical studies [25,91,92]. Most of the peptides currently approved for therapy work as agonists and are synthetic copies of naturally occurring molecules or analogs used in replacement therapy. For example, Liraglutide, commercially known as Victoza^®^, ranking 19th in drug sales (USD 4.39 billion retail), is a synthetic analog of human glucagon-like peptide 1 (GLP-1) used as a drug to manage type II diabetes (T2DM) but with a longer half-life compared to GLP-1 [93,94,95,96]. Other examples include Oxytocin [97], and Parathyroid hormones [98] and their related peptides used to induce or increase labor and for the treatment of osteoporosis, respectively [99]. Many other peptide analogs work as blocking agents, including the peptidomimetic Icatibant, which is an effective and specific antagonist of bradykinin B2 receptors, used for the treatment of acute attacks of hereditary angioedema in adults with Deficiency of C1-esterase inhibitor [100] and Saralasin, an octapeptide analog of angiotensin II (bovine), which is a highly specific competitive inhibitor of angiotensin II used in the diagnosis and treatment of hypertension [101]. An example of peptide-derived enzyme inhibitors is Enapril, an antagonist of angiotensin converting (ACE) enzyme used as an antihypertensive drug [102]. Only a few examples of marketed peptide-based drugs working as protein–protein disruptors are known. Among these, we mention the natural peptoid cyclosporine [103], an immunosuppressant drug used in organ transplantation to prevent rejection, and the antiviral Enfuvirtide, a gp41-derived peptide, which inhibits the fusion of the viral envelope and the host cell membrane [104]. A large number of peptides containing the RGD recognition motif and showing promising activity as PPIs that are currently under development as anti-thrombotics [105], and others, developed as Bak-BH3 analogs able to enhance apoptosis through the inhibition of Bcl-xL, are used as anticancer drugs [106]. Additionally, a stapled peptide named ATSP-7041 with a potent dual inhibiting activity against both MDM2 and MDMx, is now under evaluation in several clinical trials in p53-positive cancers [107].

The global market of peptidomimetic therapeutics amounted to about USD 25 billion in 2018 and is predicted to reach USD 57.2 billion in 2027 [108,109]. This estimation could not foresee the COVID-19 pandemic in 2019, which has had a strong positive impact on the global peptide therapeutic market with more than 21 synthetic peptides being used for the treatment of SARS-CoV-2 infection and many pharmaceutical industries registering increased revenues during the COVID-19 [110]. A detailed list of clinically used peptide- and peptidomimetic-based drugs has been reported and extensively discussed in a recent review [89].

## 5. Experimental Approaches Used for the Identification and Optimization of Peptides Able to Modulate PPI

The identification of peptides worth clinical development is the first and most important step in this field. It can be pursued using both experimental and computational methodologies. Excellent reviews describing the computational approaches have been published [109,111,112,113,114,115,116]. Here, we discuss the most common experimental approaches used to identify and optimize peptides with potential PPI modulation activity.

### 5.1. Protein Dissection Approach: Limited Proteolysis and MS Analysis

Starting from the information available on proteins involved in mutual interactions, the distinctive domains or highly conserved regions capable of adopting secondary structures playing a role in their physio-pathological activities can be identified. In one largely used approach, using primary or higher-order structural information, deletion mutants covering the domains of the target protein(s) can be designed, prepared, and tested to evaluate their involvement in PPIs [117]. However, in absence of enough homologous sequences and accurate structural data, these theoretical approaches fail and experimental data are needed. Limited proteolysis approaches coupled with mass spectrometry offer an elegant experimental option to unveil the exact boundaries of the domains that make up the proteins and the interacting regions in protein complexes. Peptides isolated in this way can then be used as probes for structural studies and as surrogates of PPI surfaces [118,119]. The limited protein proteolysis of native (folded) proteins is realized with selected enzymes, such as trypsin, chymotrypsin pepsin, streptococcal cysteine proteinase, and papain, under mild experimental conditions, for example, using diluted enzyme (e.g., from 1:100 to 1:2000 protein:enzyme, mol:mol), a low temperature and short incubation times. In mild experimental conditions, the proteolysis preferentially occurs at exposed and less structured sites of the protein, thus suggesting those sites are more protected by internal and external interactions or by the rigidity of the structure. The isolated polypeptides, which partially preserve secondary and tertiary structure elements [120,121], may thus work as potential inhibitors of the interactions involving the region they come from. Fragments obtained via limited proteolysis are therefore separated via high-performance liquid chromatography (HPLC), identified via MS, and then tested for activity. Finally, the active fragments can be cloned for expression or chemically synthesized for further studies.

**Figure 1:**
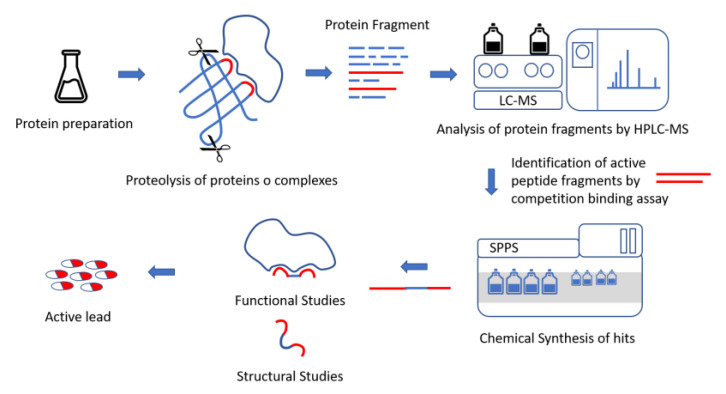
Workflow of the protein dissection approach. At first, the target protein is expressed and purified. Thus, it is subjected to proteolysis by a specific enzyme. Fragments obtained are identified by LC-MS and fractionated by semi-preparative RP-HPLC. Samples containing single peptides or known mixtures are screened as inhibitors of the target protein complex formation [120].

The workflow involves fours steps as described in Figure 1.

This technique was mainly used in the early 2000s for the identification of interaction regions in PPIs, as emerged from the huge number of articles reported in the literature [119,120,122,123,124]. Recent studies on the development of peptides and small molecules as well as of drug-like compounds have greatly benefited from this methodology. A representative example is presented by the study of the complex HSP90-CDC37. The complex between the chaperone HSP90 (Heat Shock Protein 90) and the co-chaperone CDC37 (Cell Division Cycle 37) is involved in the folding and activation of many kinases, which differ from the substrates of HSP90 [125]. In many cancers, CDC37 is overexpressed and facilitates the folding and activation of specific oncoproteins important for tumor progression [126]. Thus, the HSP90-CDC37 complex has long been considered a great antitumor target and an alternative strategy to block HSP90 activity in cancer. Indeed, the blockade of the HSP90-CDC37-kinase chaperone ring offers the option to selectively regulate HSP90 kinase substrates in alternative to inhibiting all client proteins suppressing the ATPase activity. Shao J et al. [121], to identify the interaction region between CDC37 and HSP90, fragmented with enzymes the native recombinant CDC37 under limited proteolysis conditions isolating three distinct domains. They found that the middle domain, encompassing residues 128–282, mainly interacts with HSP90 paving the way to the development of compounds against this therapeutic target [127,128,129].

This methodology finds today an extensive application for the identification of “Food-derived bioactive peptides” [130,131]. Several studies showed that biologically active peptides could be successfully isolated from seaweed [132,133]. As an example, Sun et al. (2019) identified new small inhibitory peptides of the Angiotensin I-converting enzyme (ACE), a prominent target for treating hypertension and cardiovascular diseases, from the hydrolysate of proteins obtained from the Marine Macroalga Ulva intestinalis [134].

The limited proteolysis experiment directly carried out on protein complexes will indicate the protein subunits involved in the interaction, as molecular surfaces involved in the interaction are more protected from proteolysis. The data obtained from these studies can be also used for validating the assembly mode of protein subunits in a given complex and to provide an overall evaluation of the complex topologies. In one recent work [135], we probed the accessible surfaces of CypA in the presence and absence of a biologically active peptide derived from AIF (AIF (370–394)) to support NMR results and provide further experimental evidence of the interaction surfaces with AIF. The CypA/AIF complex has a pro-apoptotic activity and is involved in oxidative stress-mediated pathologies, such as neurological and cardiac diseases [136,137,138]; therefore, knowledge of the recognition interface is crucial for developing inhibitors. The results of this study provided significant evidence of the interaction between the peptide and the protein and contributed to elucidating the segment of CypA contacted by AIF, in line with NMR and other molecular analyses. The new peptide identified binds AIF with a KD in the low micromolar range [135].

### 5.2. Combinatorial Approaches

A valid experimental approach for discovering peptide-based PPI modulators is the combinatorial approach. In this approach, many different molecules are prepared in a single process and screened for activity in high-throughput screening (HTS) assays. The overall approach integrates several disciplines, including molecular biology, synthetic and computational chemistry, analytical methodologies, molecular modeling, and HTS. Depending on the strategy, combinatorial peptide libraries can be tuned in multiplicity (up to several millions of different molecules) and diversity to accommodate a wide range of different peptide structures or to explore well-defined and limited binding pockets or binding surfaces with small-sized and focused ensembles of peptides. Essentially, peptide libraries are classified as synthetic or genetically encoded, depending on the method used to produce them.

#### 5.2.1. Phage Display

**Figure 2:**
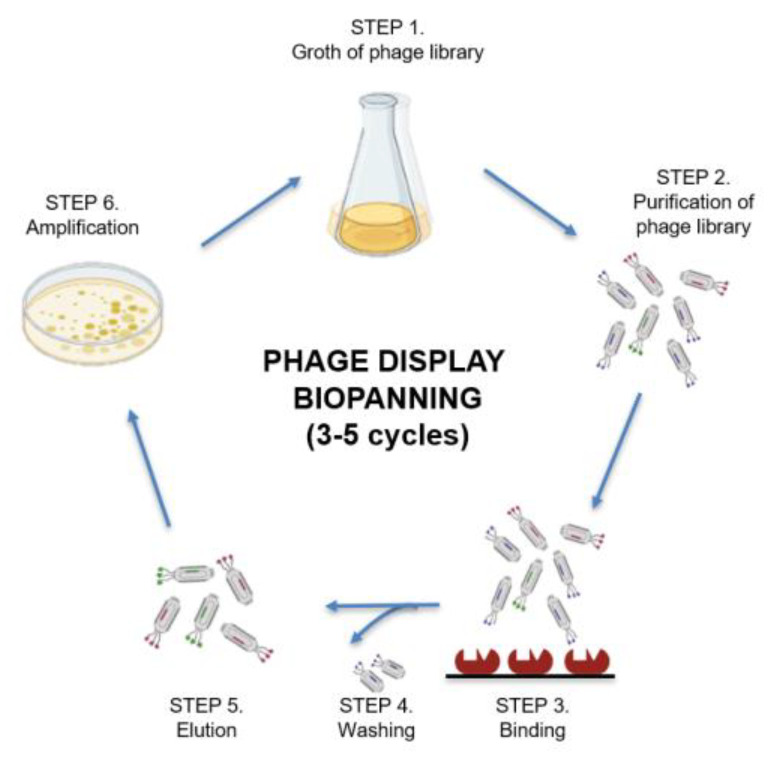
Schematic representation of the phage display technique. In the first step, the library is built and amplified (STEP 1). Then, it is purified (STEP 2). After purification, the library is incubated with a target molecule to allow for binding (STEP 3). With subsequent washings, unbound phages are removed (STEP 4). In the “elution step”, phages bound to the target are detached via pH change or via competitive elution and collected (STEP 5). Eluted phages are used to infect bacteria to amplify them following grow and re-purification (STEP 6). This creates a new and more selective phage sub-library that is used in a subsequent round of biopanning. The picture is inspired by ref. [141].

Phage display libraries are genetically encoded peptide libraries. They have long been used to identify ligands of biological targets, especially for membrane receptors. They were first used by George Smith in 1985 [139] and the first patent was filed in 1991 [140]. In this approach, recombinant technologies are used to express polypeptides of various lengths and complexity on the surface of bacteriophages in a way that every single virus particle presents a peptide with a different sequence. The exposed protein fragments are screened for their binding ability toward a target biological target through an affinity selection procedure [141]. The whole workflow is shown in Figure 2.

Many ligands for relevant receptors, such as cell adhesion molecules and oncogenic proteins, have been identified using this technology [141,142,143,144,145,146,147,148]. The main advantage of phage display is the ability to quickly produce massive libraries up to 10^11^ compounds [149], which are easily replicated and amplified by culturing the phage colonies. Libraries are also simply screened using binding tests and errorless sequencing of the bound phages. A chemical library also enables the generation of huge libraries in the form of mixtures; however, the screening process is more complex requiring elaborate deconvolution processes [149]. However, the phage display technology has several drawbacks, the most important of which is the inability to work with peptides containing non-proteinogenic amino acids. Additionally, the lack of control during the rounds of biopanning may lead to poor enrichment degrees and modest specificity of the final ligands. Some limitations have been overcome by recent advancements of screening protocols and by the introduction of post-translational chemical modifications to generate, for example, mono- or poly-cyclic peptides [150,151,152,153]. Relevant reviews on this topic can be found in Ledsgaard and Ljungars’ 2022 [154] and Jaroszewicz et al.’s 2021 work [155].

Aillaud et al. have recently reported a highly representative example of the use of phage display technology. They selected a peptide against the Tau full-length protein (TauFL) acting as a potential inhibitor of the early stages of the pathological fibrillation cascade. In the first step, an L-peptide bind TauFL was selected. The D-amino acid (named ISAD1) and the backbone inverted (named ISAD1rev) variants were next designed and prepared via chemical synthesis, thus finding that the new molecules inhibited fibrillation of TauFL and of several Tau variants associated with the disease [156].

#### 5.2.2. Chemical Synthesis of Peptide Libraries

Combinatorial chemistry methods enable the ad hoc chemical synthesis of large numbers of compounds designed in parallel or serial processes leading in a relatively short time to large collections of single or mixtures of compounds named molecular libraries. The size of a library can range from a small set of peptides (<100) to thousands or even millions of different chemical species produced using the fully optimized solid phase synthesis protocols. Screening of such collections without isolation or purification of individual compounds is iteratively achieved through assays that measure biological activity and make it possible to identify individual compounds capable of altering it. Combinatorial chemistry also offers an efficient route for the structural optimization of peptides by generating arrays of derivatives of a parent molecule. Unlike the methods involving phage display expression and limited proteolysis, the approach of synthetic peptide libraries is much faster and prevents the loss of potentially active sequences.

The combinatorial peptides libraries approach has been largely used for: (1) target validation; (2) drug discovery and development; (3) epitope mapping; and (4) structure–activity relationship studies [157,158].

The synthetic methods for peptide combinatorial libraries are based on the solid-phase peptide synthesis (SPPS) invented in the 1960s by Merrifield [159]; however, the first concepts of combinatorial chemistry were developed using Houghten’s tea bag and Geysen’s multi-pin technologies in the mid-80s used for the synthesis of hundreds of thousands of peptides in parallel on solid supports [160,161], and further with the introduction of the split and mix method associated with OBOC (one-bead–one-compound combinatorial peptide) libraries and the SPOT synthesis on cellulose [162,163].

Large random peptide libraries are mostly generated as mixtures of compounds via methods such as the “pre-Mixing” and OBOC approach. The preparation methods of the peptide libraries are beyond the scope of this work; therefore, for an exhaustive description of them, please refer to the following papers reported in the literature [162,163,164].

It is worth underlining that unlike small molecules, for which public and commercial databases of both bioactive and “unexplored” structures are available, due to ease of designing and preparing peptide libraries, only databases of bioactive peptides are available, such as BIOPEP [165] and DFBP [166].

Regardless of the preparation method, there are at least six types of synthetic peptide libraries that differ from each other in design and purposes. These are: (a) overlapping peptide libraries, (b) truncating peptide libraries, (c) peptide scanning libraries (d) alanine scan libraries, (e) positional or scrambled peptide libraries, and (f) peptidomimetic libraries.

**Figure 3:**
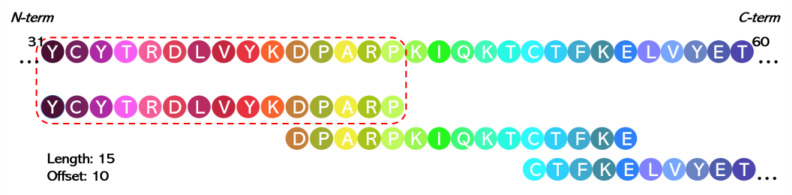
Simplified representation of overlapping synthetic peptides used to map the different interaction regions in the FSH-β subunit. The peptide FSH-β-(31–45) identified in this study, shown in the red circle, included the region FSH-β-(34–37), previously identified as a receptor-binding region [174].

**Figure 4:**
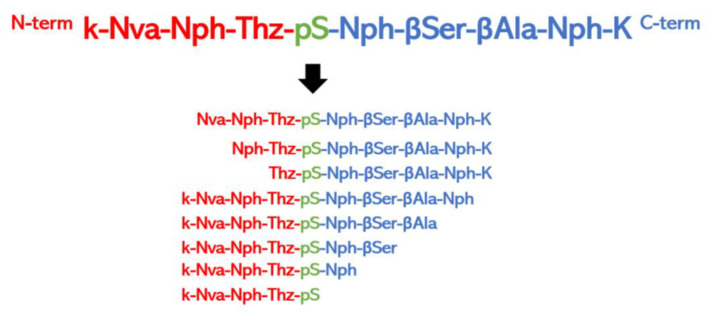
Sequence of 14-3-3 binding peptide (upper) and of N- and C-terminal truncated peptides designed starting from it to obtain a 14-3-3 PPI stabilizer [181]. pS denotes the phosphorylated-Serine residue.

(a) Synthetic overlapping peptide libraries (OPLs). Overlapping synthetic peptide libraries are generated by dividing the original protein or domain sequence into many overlapping peptides of the same or similar length. It has been commonly used for epitope mapping generating peptides with lengths between 8 and 20 amino acids with an offset of 5 to 10 amino acids [167,168,169,170,171,172,173]. In a recent work [174], starting from the 3D structures of FSHR::FSHβ [175] and FSH::FSH [176] and from previous studies, a library of 15-mer overlapping peptides (the library is composed of 11 single peptides), with 5 overlapping amino acids at each end (Figure 3), was used to map the receptor-binding regions of FSH-β as well as the contact regions between the FSH α and β subunits. The results corroborated previous data obtained with synthetic peptides and are in line with the crystallographic structures, demonstrating the validity and utility of this approach.

(b) Truncation peptide libraries. Truncation peptide libraries are commonly used to identify the shortest region of a peptide required for a given activity [177]. The library is constructed by systematically shortening the N- and C-terminal residues of the original peptide. Many very short bioactive peptides have been reported in the literature [178]. For example, the small peptide YGGFM, which is part of the Met-enkephalin, retains the full activity of β-endorphin, an endogenous opioid of 3465 Da [179]. Similarly, the four residues C-terminal peptide WMDF-NH_2_, known as CCK-4 obtained from the larger peptide hormone cholecystokinin, is the shortest portion of the parent peptide that retains full biological activity [180]. Therefore, this approach can give significant help in simplifying a starting peptide and maintaining its activity. For example, Somsen et al. in 2022 [181] performed a functional mapping of the 14-3-3 protein in complex with a peptide ligand to arrive at a peptide-based so-called molecular glue for the 14-3-3/ChREBP protein complex. A hotspot analysis was performed by truncating and mutating the original peptide, providing important mechanistic information on the protein binding of the 14-3-3 binder (Figure 4).

(c) Peptide scanning libraries. Peptide scanning is based on the design and synthesis of fragments covering a protein sequence or large parts of it. The binding to the respective partner protein is then tested with the individual peptides to select potentially active ones [182]. The library may be designed to contain either peptides that mimic the solvent-accessible surface of the protein or selected protein domains or the full-length protein [182,183]. This method makes it possible to identify protein-binding sites, but only in cases where they consist of continuous and sequential stretches and there are no contributions from distant but three-dimensionally closed regions. Compared to limited proteolysis or the expression of deletion mutants, this approach allows for a faster and more cost-effective assessment of the interaction interfaces involved in PPIs.

**Figure 5:**
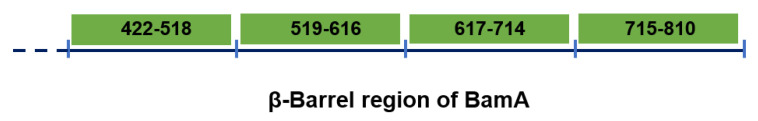
Schematic representation of peptide designed on the β–Barrel domain of BamA [185].

The correct assembly of proteins in the outer membrane of Gram-negative bacteria takes place through the β-barrel assembly machine (Bam) [184]. In the Bam multiprotein complex, one component, BamD, interacts with unfolded protein substrates, such as BamA, promoting their assembly in the outer membrane [184]. Hang et al. [185], using a peptide scanning approach identified a 15-mer peptide from the C-terminal region of BamA able to inhibit the assembly of outer membrane protein (Figure 5). Expressing in vivo this peptide impaired bacterial growth and sensitized Escherichia coli strains commonly resistant to antibiotics. The study suggested that the synthetic peptide might be used as the template for generating new antibiotics against Gram-negative bacteria.

(d) Alanine scanning library. In alanine scanning library, the residues of a peptide are systematically replaced with an alanine generating a different peptide for any position changed in the original peptide. Testing the biological activity of these analogs provides insights into the relevance of specific residues and uncovers structural determinants of the peptide-protein recognition interface. Using this approach, tolerable alanine residues and irreplaceable side chains working as “hotspots” are identified. In a subsequent step, new analogs are designed to try to find the best active peptide, introducing new side chains or modifying the peptide backbone [186]. This approach found enormous application in the early 2000s; today, however, in silico approaches are extensively used. For example, on an inhibitor of Bcl-xL, a protein involved in the apoptosis process [106], the sequence G1-Q2-V3-G4-R5-Q6-L7-A8-I9-I10-G11-D12-D13-I14-N15-R16 was investigated as regards performing alanine scan, obtaining clear experimental evidence of residues important for the biological activity. Indeed, L7 was shown to be a hotspot, as its replacement with alanine produced the most dramatic reduction in biological activity. In contrast, residues I9, G11, and D13 were not important, as their substitution did not affect ligand affinity [106]

**Figure 6:**
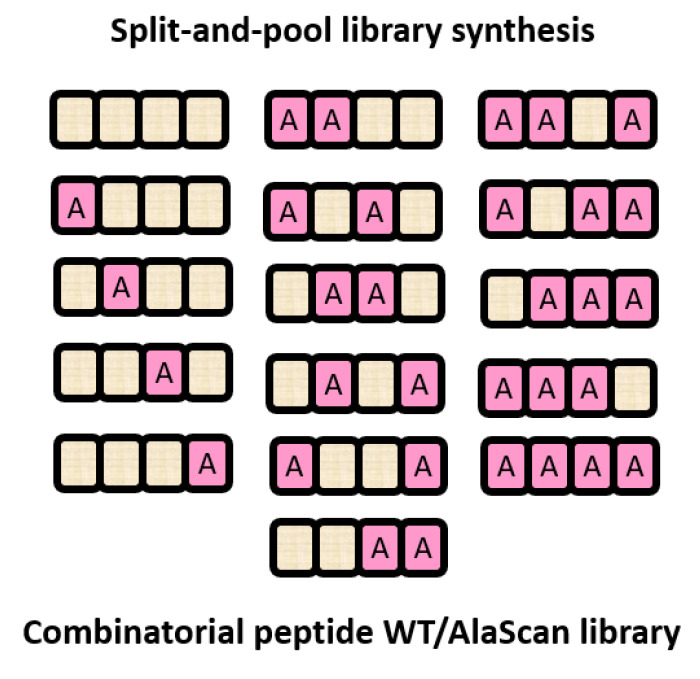
Schematic representation of binary combinatorial scanning design [187]. The empty squares indicate the fixed amino acid positions, while the others (containing the “A”) the positions where the amino acids have been mutated with the alanine residue.

Traditionally, alanine scanning is performed modifying a single residue at a time. However, single alanine mutations may not be effective and multiple replacements may be required to reveal the complete SAR landscape of a peptide. To overcome this limit, Ye et al. in 2022 validated a method for the optimization of peptide-based binders [187], using combinatorial peptide libraries of over 4000 variants, where each position and multiple positions were simultaneously varied with alanine. Peptide libraries were synthesized via the split-and-pool method and tested with a label-free method. The peptide library was exposed to the target protein, which was subjected to high-performance size exclusion chromatography (HPSEC) to separate unbound ligands from those bound. The bound peptides were identified via liquid chromatography-tandem mass spectrometry (LC-MS/MS). The applicability of this platform was evaluated by testing the library against the oncogenic protein MDM2, the 12ca5 antibody and the 14-3-3 regulatory protein [187]. From these studies, several multi-alanine-substituted analogs with binding affinities in the pM range were discovered for the systems studied demonstrating their general applicability (Figure 6).

(e) Positional scanning (PS) libraries. Positional scanning (PS) libraries are important tools for peptide sequence optimization. In this approach, the peptide library is divided into sub-libraries, each consisting of a mixture where one fixed position is defined by the presence of a known amino acid, while other amino acids are incorporated as mixtures. All sub-libraries are analyzed simultaneously for activity to identify the consensus peptide sequence with the best activity in the chosen screening assay [164,188]. In its original format developed by Furka and colleagues, the bioactive peptide sequences are decoded iteratively one residue at a time by preparing sub-libraries and performing a screening assay for each sequence position. The single positions are identified gradually using an iterative deconvolution strategy where residues defined in the first N-terminal position are kept in the next-generation sub-library, which contains the next residue defined in the second position. The approach is thus repeated until all positions are queried [164,189].

**Figure 7:**
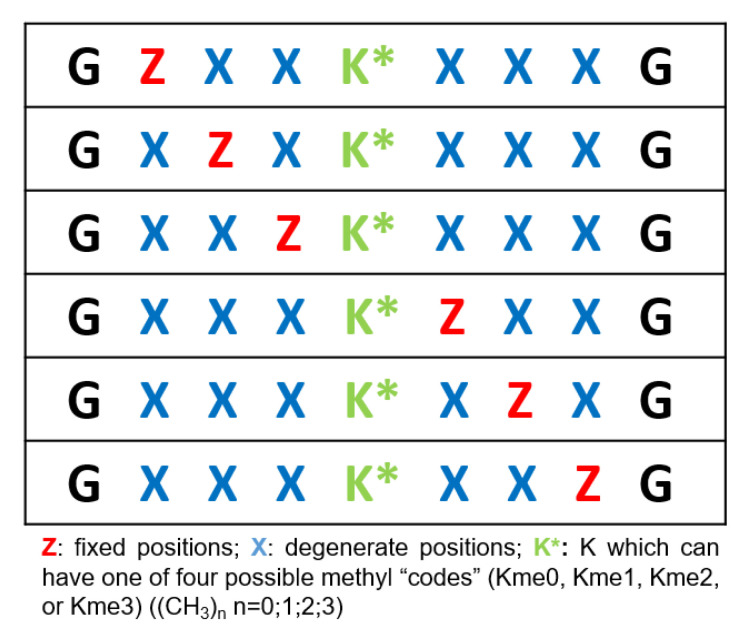
Representation of Kme-OPL library. The Kme-OPL contains 114 sets of 9-mer peptides formatted into 6 sub-libraries featured by a fixed central lysine. Each sub-library contains 19 sets of nonapeptides, one set for each different amino acid used. The remaining positions represent an equal mixture of all amino acids [190]. G denotes a glycine residue.

For example, Kupai et al. (2020) developed a degenerate methylated lysine-containing peptide library (Kme-OPL) to detect the code of methyl group positions required for the recognition of multiple Kme (Lysine methyltransferases). The study also aimed at unveiling the consensus sequence “read” at best by the Kme and revealing the recognition elements of histone Kme specific antibodies [190]. Peptide libraries were generated in PS format [191]. They also developed a Kme-OPL reader platform based on magnetic beads used in pulldown assays where the readout was fluorescence intensity. A set of known ligands including histone Kme-specific antibodies, was used in the pulldown assays to validate the platform. The library was organized around a central lysine residue, which can have one of four possible methyl “codes” (Kme0, Kme1, Kme2, or Kme3) (Figure 7). In every code, the library is formatted into 19 *×* 6 = 114 Kme-OPL sets, with each set having a fixed amino acid at a given position (six positions), while the others are filled with equimolar mixture of 19 amino acids (excluding cysteine). The peptides are biotinylated to allow their immobilization on the magnetic beads containing the streptavidin. The sets of magnetic beads and Kme-OPL are first loaded with the libraries, and then a ligand of Kme conjugated to a recombinant GST tag is added. Next, a primary GST antibody is added followed by a fluorophore-conjugated secondary antibody. Binding is detected via fluorescence intensity measurements. Data obtained by this study are in line with previous reports showing similar structures in the CDYL2 Kme peptidomimetic inhibitor, UNC4991 [192].

**Figure 8:**
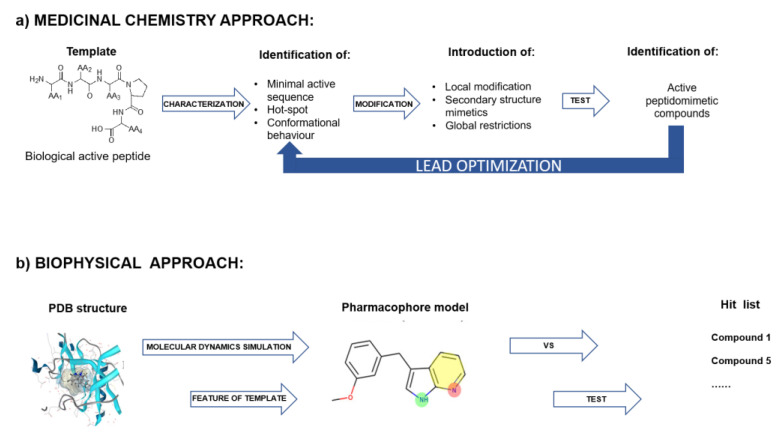
Approaches for the design of peptidomimetics: (**a**) the medicinal chemistry approach; (**b**) the biophysical approach.

(f) Peptidomimetic libraries. Various strategies have been developed to position the chemical functions of the side chains as in the parent peptides to obtain new “depeptidized” variants or peptidomimetics [193]. Essentially, there are two different approaches to the design of peptidomimetics and peptidomimetic libraries. In one approach, known as the medicinal chemistry method, selected parts of the bioactive peptide are replaced by non-peptide moieties so that improved pharmacokinetic properties are achieved. In a second approach, known as the biophysical method, peptidomimetics are designed “ab initio” starting from the putative bioactive form of the peptide and using different scaffolds decorated with appropriate functionalizations (Figure 8a,b) [194,195].

Using synthetic peptides to mimic protein regions in their bioactive conformation has long been a common practice for the design of PPI inhibitors and protein binders with improved stability features compared to “pure” peptide precursors. Cyclization is among the more frequently applied strategies to rigidify peptide structures and stabilize bioactive conformations. Peptide chains are commonly cyclized by head-to-tail amidation, disulphide bond formation between cysteines, side-chain amine to C-terminal carboxyl group, and side-chain carboxyl group to N-terminal amidations [88]. More recently, peptide cyclization has been revolutionized by the introduction of non-canonical orthogonal reactive groups able to give selective and quantitative reactions [88]. The introduction of these elements in cyclic peptides has expanded, in addition to the chemical space of the precursors bringing significant structural diversity, which might be exploited to generate new libraries and more potent ligands [88]. The combination of these different approaches has by far contributed the most to the rapid development of cyclic peptide drug discovery [92].

Peptidomimetics and their libraries can also be designed by generating retroinverso analogs of the parent peptides [196,197]. Retroinverso analogs showing the same activity as the precursors have the main advantage of longer half-life lasting due to resistance to proteases. A representative example is the totally retro-inverted analog of OR2, a peptide that has shown promising results for the treatment of Alzheimer’s disease [198]. OR2 is a decapeptide with inhibitory activity against beta-amyloid aggregation, identified by screening a set of peptides designed on the beta-amyloid sequence [199]. To find peptide surrogates of OR2 with better pharmacokinetic profiles, the retro-inverted analog RIOR2 was designed and synthesized, which proved to be as active as the starting molecule but much more stable to proteolysis [198]. Given its improved pharmacological profile, this analog will allow continuing further development to demonstrate the efficacy for the treatment of Alzheimer’s disease [200].

The biophysical approach makes use of scaffolds holding the chemical groups involved in ligand–receptor recognition (pharmacophoric groups) in the appropriate orientation [194]. The approach assumes that only side chains play a role in the interactions with the target receptor and the resulting structures have often been reported as secondary structures mimics. However, many “side-chain only” peptidomimetics do not remain frozen in single conformational states; however, these may easily adopt several freely interconvertible conformations which can resemble various secondary structures, in some cases referred to as round or helical mimics understates, depending on how they adapt into various binding situations. Sets of scaffolds can be constructed to mimic the organization of almost any secondary structure, thus generating peptidomimetics from any kind of starting conformation. These can be the methods of choice in library design, particularly when the binding conformations of the ligands sought, or even of the target, are unknown at the time of library design [201]. In 2012, Whitby and Boger built three libraries based on certain templates designed to resemble each secondary structure element and replaced them with all the triplet combinations of functional groups found in the 20 natural amino acid side chains so that they could contain at least one member capable of mimicking the crucial interaction residues of most targetable PPIs. They screened an α-helix mimetic library against the p53/MDM2 and HIV-1 gp41 complexes, while using a β-turn mimetic library to screen the opioid receptors. The study led to the discovery of library members predicted to mimic the endogenous ligands, showing that the approach is a powerful method for interrogating PPIs [202]. Using the same approach, an antitumor peptide designed based the protein–protein interaction interface of myc and its retroinverso analog were identified and showed significant antiproliferative activity. In another study, starting from the retroinverso analog of the core amyloid peptide (KLVFF), three generations of peptidomimetics were developed by replacing the natural amino acids with non-coded amino acids, identifying a peptide effective in preventing amyloid fibril formation and in reverting to soluble forms those already formed in models of Alzheimer’s disease [203].

## 6. Successful Applications of Synthetic Combinatorial Approaches in Human Diseases

Here, we review significant historical studies and very recent discoveries of peptide hits and peptides used as drugs or with promising perspectives of treating various human diseases, made using libraries of synthetic combinatorial peptides.

### 6.1. Neurological Diseases

Neurological disorders (NDs), including Alzheimer’s and Parkinson’s diseases, strokes, multiple sclerosis, epilepsy, brain injuries, and neuroinfections, affect one in six of the world’s population. Up to 6.8 million people die from these diseases every year [204]. Treatments available to manage NDs are still insufficient as the high failure rate in clinical trials strongly limits the number of approved drugs in this field and there is a continuous demand for new drug candidates to cure NDs. Combinatorial peptide library screening is an approach widely used in the drug discovery process for new chemical entities against NDs.

In several human neurodegenerative diseases, the triggering factor is protein misfolding accompanied by aggregation phenomena [205], which is the root cause of Parkinson’s (PD), Alzheimer’s (AD), Huntington’s (HD), Prion diseases (PrD), cystic fibrosis, amyotrophic lateral sclerosis (ALS) and others [206].

The prominent neuropathological feature of AD is the amyloid deposition in the brain parenchyma and cerebrovascular system of a peptide called Aβ [207,208,209]. This peptide is a 40 to 42 amino acid long proteolytic fragment excised from the Alzheimer’s amyloid precursor protein (APP), an almost ubiquitous protein [210]. A promising approach for treating AD is based on the inhibition of aggregation of Aβ [208,209]. In this framework, Tjernberg et colleagues in 1996 [211] identified the Aβ(16–20) internal fragment, of sequence KLVFF, as a potential aggregation inhibitor. They designed and prepared an array of 10-mer overlapping peptides, for a total of 31 peptides, on a cellulose membrane matrix using the SPOT technique and identified the central region of Aβ spanning residues 9–22 as the fragment mostly involved in the self-assembly and consequent aggregation of Aβ. Following the same approach, they prepared and screened a set of N- and C-terminally truncated peptides, identifying the shortest fragment with the desired activity, and finally through an alanine scanning approach they defined hotspots responsible for activity [212]. Notably, this peptide has been used in many other studies as a template for generating peptide-based inhibitors of Aβ fibrillation [213,214,215,216,217,218,219,220]. As an example, very recently, KLVFF has been conjugated with nanomagnets based on amphiphilic cyclodextrin for the selective recognition and capture of soluble Aβ(1–42). These nanomagnets have the potential to early diagnose Alzheimer’s disease using the fluids of patients [221].

The anti-amyloidogenic activity of the peptide fragment Aβ(1–6) conjugated to the cell and BBB penetrating peptide, named Aβ(1–6)-A2V-TAT (D-GRKKRRQRRR-GGGG-DVEFRH) was investigated in vivo [222]. The original N-terminal fragment of Aβ was mutated in position 2 from alanine to valine acquiring a strong anti-amyloidogenic activity in vitro and a potent ability to inhibit Aβ aggregation in mouse models of Alzheimer’s disease [223]. Luo et al. constructed an on-bead peptoid library of 38,416 compounds, to obtain selective high-affinity ligands for Aβ(1–42) [224]. The compound identified, named IAM1, and its dimeric form (IAM1)2, significantly inhibited Aβ(1–42) aggregation in vitro. The dimeric peptide also protected primary hippocampal neurons from Aβ(1–42)-induced toxicity. Using a one-bead-one-peptide combinatorial approach Richman et al. identified the cyclic peptide CP-2 from the screening of a 6-residue cyclic library of 7776 member, obtained from head-to-tail closure of d,l-α-peptides containing the residues K, E, S, L, W and H [225]. The selected peptide strongly binds to both Aβ(1–40) and Aβ(1–42), preventing their assembly and protecting PC12 cells from the toxicity induced by Aβ(1-40)/Aβ(1–42) without toxic effects.

Bartling et al. in 2021 [226], sought new strategies to treat AD by modulating the interaction of direct binding partners involved in APP trafficking and processing. They first performed a deep characterization of the interactions of APP with Mint2 involved in Aβ formation. Then, generated and tested N- and C-terminal truncated variants of the 17-mer human peptide of APP (QNGYENPTYKFFEQMQN; residues 754–770 in APP) identifying a 12-residue-long peptide, designated as APPWT (NGYENPTYKFFE; residues 755–766; Ki = 4.0 ± 0.2 μM) as the minimal sequence involved in Mint2 binding [226]. Through a systematic alanine substitution approach, they also identified the residues crucial for the interaction and developed a high-affinity, proteolytically stable cyclic peptide made of D- and N-methylated amino acids. Zhang et al. (2022) [227] used a PS library to develop selective activity-based probes (ABPs) and inhibitors of the kallikrein-related peptidase KLK6. KLK6, a serine protease, orchestrates the so-called KLK activome, a protease cross-activation network strongly connected with neurodegenerative diseases, skin disease, and cancer [228]. However, the lack of compounds against KLK to profile its activity has largely hampered its validation as a putative target or biomarker for these diseases. In this article, by a PS Substrate Library and an iterative process authors developed a set of optimized peptide-based compounds as probes and inhibitors of KLK6, which allowed for the first-time the evaluation of the activity and specificity of KLK6, and provided significant insights into KLK6-mediated invasion pathways. Four new libraries, containing each 106 individual sub-libraries with 361 peptides were generated, covering 114,798 different combinations. Data also offered a valuable tool not only for validating the role of KLK6 in PDAC but also for identifying associated signaling partners as targets or biomarkers in a large range of diseases.

In recent years, research data indicated there is a strict connection between proteins involved in AD and type 2 diabetes (T2D) that form β-sheets such as motifs. This hypothesis finds evidence in the misfolded IAPP produced in T2D that can potentiate AD pathology by cross-assembling with Aβ and providing a molecular link between these diseases [229,230,231]. IAPP is a neuropeptide that, in soluble form, has a glucose regulatory role. It co-localizes with Aβ plaques in AD patients’ brains, suggesting a potential pathophysiological role of this cross-interaction [231,232,233]. To understand how misfolded IAPP affects Aβ aggregation, a systematic alanine scanning approach together with biological assays and in silico analysis were applied by Kapurniotu’s team to identify key molecular determinants of the cross-interaction of IAPP with Aβ. They identified the single aromatic/hydrophobic residues used by IAPP amyloid core region to interact with Aβ, but not to self-assemble [234]. The main bottleneck for the treatment of most degenerative diseases, including AD or glioblastoma multiforme (GM), is the penetration of the BBB which prevents the diffusion of toxic foreign substances in the brain parenchyma but also blocks the delivery of therapeutic drugs to other brain regions. Recently, applying the OBOC concept a combinatorial chemical library was screened to identify a peptide targeting the epidermal growth factor receptor (EGFR) which is overexpressed in almost 50% of GM patients [235]. They prepared and screened a 10-mer OBOC peptide library labeled with SA-coated magnetic beads and separated them in a magnetic microfluidic chip, unveiling the active peptide sequences by in situ MALDI-TOF-MS. To identify the most active peptide, they redesigned a consensus 5-member library elongated with a cysteine residue for the capture on a gold chip for subsequent screening through surface plasmon resonance imaging (SPRi). The OBOC combinatorial library had general sequence X1X2X3X4X5X6X7X8X9GM. Each X represented any of the four different selected amino acids, described as follows, to improve the diversity of the library. X1 stood for F, W, R or K, X2 for V, D, K or E; X3 for K, F, L or R; X4 for E, V, R or Y; X5 for L, E, F or Y; X6 for Y, K, D or V; X7 for E, L, W or R residues; X8 for E, R, F or D; and X9 for Y, K, V or L. With this study authors developed a dual-targeting drug delivery system based on the fourth generation PAMAM dendrimer conjugated with the identified EP-1 peptide and the angiopep-2 peptide (Ang2, TFFYGGSRGKRNNFKTEEY), a sequence that could induce BBB transport through binding to LRP1 [236].

### 6.2. Cancer

Cancer is the leading cause of death worldwide. The total number of cancer-related deaths is far higher than that of other diseases [237].

PRMT5-MEP50 methyltransferase is an increasingly important target for developing anticancer drugs. Modulators of its interactions with various regulatory proteins are continuously sought as they modulate the selectivity of PRMT5 for substrates. Krzyzanowski et al., in 2022, proposed a strategy to identify inhibitors of the interaction of PRMT5 with adapter proteins. It was based on the design and synthesis of macrocyclic peptides able to inhibit the interaction of PRMT5 with RioK1, one of its adapter proteins. Macrocycles of different sizes and containing various cyclization linkages were first evaluated. Then, through the screening of small peptide libraries (10 peptides in the first-generation library and 17 in the second-generation library) that explored various amino acid structures, they identified binding hot-spots which once replaced with non-proteinogenic amino acids led to a cyclic peptide with a potent and selective inhibition activity (Ki = 66 nM) of the binding of PRMT5 with RioK1. The inhibitor is a promising candidate for further biological investigations on this clinically attracting protein interface [238]. Jiang et al. [177] designed and synthesized a series of high-potency peptidomimetics (for a total of 50 peptidomimetic inhibitors), that efficiently blocked the interaction between adenomatous-polyposis-coli (APC) and its receptor Asef, both in vitro and in colorectal cancer cells. Following a structure-based drug design approach they created an array of peptidomimetic that inhibited the APC-Asef PPI. Yang et al. [239] starting from the crystal structure of APC complexed with a binding peptide (Z-^181^AGEALYE^187^-NH_2_) [177], found that the substitution of A184 with a serine led to a new peptide called MAI-400 peptide showing an affinity of 0.012 μM and an IC50 of 0.25 μM. The key role played by the new residue in the formation of an intramolecular hydrogen bond crucial for the high affinity was shown by surface plasmon resonance, X-ray crystallographic analysis, tryptophan fluorescence, and ITC.

The Keap1/Nrf2-ARE (Kelch-like ECH-associating protein 1) antioxidant stress signaling pathway plays a crucial role in a variety of oxidative stress-related diseases including cancer [240]. Under physiological conditions, Keap1 targets Nrf2 to initiate the ubiquitin-controlled degradation of proteins. Upon oxidative stress, Nrf2 evades degradation mediated by Keap1 and enters the nucleus where activates the expression of antioxidant and cytoprotective genes [241,242]. Most inhibitors of the Keap1/Nrf2 interaction act by covalently attacking the sulfhydryl group of cysteines in Keap1, leading to oxidation or alkylation reactions that, inducing a conformational change in Keap1, prevent the interaction with Nrf2 [243]. The long-term use of this kind of Keap1/Nrf2 inhibitors enables the accumulation of active Nrf2, which may trigger other mechanisms leading to cancer [244]. To overcome these side effects, the use of non-covalent inhibitors of the Keap–Nrf2 interaction is therefore becoming an urgent therapeutic need [245]. The 3D structure of the complex is available [246] and has prompted the development of peptide inhibitors [247,248,249,250,251]. In studies by Georgakopoulos et al. [247], libraries of fluorescent peptide probes were tested, identifying a peptide with inhibitory activity against the Keap1/Nrf2 interaction. Further modifications of one selected peptide in terms of length, N-terminal groups, and presence of fatty acids or of the TAT cell-penetrating peptide, led to compounds with largely increased activity [248,249,252,253].

To pharmacologically antagonize the MDM2/X-p53 (hDM2/X-p53 in human) interaction, involved in many cancers, several small-molecules have been developed, such as Nutlin-3 and its derivatives [254,255,256,257]. A limitation of these molecules is associated with their inactivity against MDMX, the major inhibitor of p53 [254,255,256,257,258]. To fill this gap, active peptides were selected from combinatorial libraries designed based on the p53-MDM2/X binding site (ETFSDLWKLLPE). Among the others, peptides named 12/1 (sequence MPRFMDYWEGLN) [259], _ENREF_17PMI (sequence TSFAEYWNLLSP) [260], and pDI (sequence LTFEHYWAQLTS) [261] have been reported. The peptide pDI has been further developed to improve its kinetics and dynamic properties by screening arrays of stapled variants [262,263,264,265]. As an example, Bernal et al. successfully applied this strategy to identify a 16-residue α-helical peptide derived from p53 (SAH-p53-8) stabilized by a covalent bridge. The peptide exhibited a high affinity toward both MDM2 and MDMX [266], providing a proof-of-concept of the potentiality of concomitant inhibition of MDM2 and MDMX. Despite its constrained structure, the new peptide proved to be of limited biological interest because of its instability in pseudo-physiological conditions. Through an iterative lead optimization process, Chang et al. [107] optimized pDI and SAH-p53-8, devising a new stapled peptide named ATSP-7041, which preserves the biologically active α-helical conformation of the precursors and possesses favorable drug-like properties, including high-affinity for both target proteins, efficient cell penetration and excellent in vivo stability. ATSP-7041 has shown a therapeutically promising activity profile in p53-positive tumors driven by MDM2 and MDMX. Libraries of synthetic stapled peptides have been also successfully used to identify modulators of other therapeutically relevant targets for cancer therapy, such as BH3, Notch, Hif-1, α,β-catenin, and Ras [267,268,269,270].

### 6.3. Infectious Diseases

Viral infectious diseases are emerging as one of the most dangerous public health burdens that can quickly lead to millions of deaths. Despite the huge efforts made to treat and prevent viral infections, the complex life cycle and uncontrolled genetic mutations of viruses drive the continuous development of new, safer, and more effective drugs. Peptides have the prospect to become tools to combat the spread and recurrence of viral infections and synthetic peptide libraries can be the valuable basin from which new synthetic antiviral or antimicrobial agents for the control of infectious diseases can be isolated.

In this field, pioneering studies have been conducted to develop NCE against HIV-1 infection. Most anti-HIV drugs target the viral reverse transcriptase and protease enzymes. Several concerns have been however raised regarding both the long-term efficacy and the ability to overcome viral resistance to this class of drugs. Many efforts have been put into blocking HIV-1 infection at later stages of the virus life cycle, such as viral cell attachment and entry. Entry of HIV-1 into host cells originates from a cascade of PPIs events involving the trimeric viral spike, composed of glycoproteins gp120 and gp41, and the major host receptor CD4 and coreceptors CCR5 and CXCR4 on the host cell [271]. Using synthetic peptide libraries, several antagonists blocking HIV-1 infectivity have been developed, including linear or structurally constrained synthetic peptides [272,273,274,275,276,277,278,279]. Wild et al. in 1992 [278] developed a series of peptide antagonists of HIV-1 based on the amino acid sequence of gp41 (for example, DP107 and DP178, also known as T20). Using truncated and mutational peptide scanning libraries [280], the mechanism of inhibition of the T20 peptide was clarified at the molecular level [104]. To date, the peptide T20 (generic name: Enfuvirtide, trade name: Fuzeon) is the only FDA-approved HIV fusion inhibitor clinically used to treat HIV/AIDS patients unresponsive to current antiretroviral drugs. However, the T-20 therapy requires a high drug dosage that may likely induce drug resistance. Recently, Chong et al. have reported a series of short-lipopeptide-based fusion inhibitors, identified from a large library of single peptides conjugated with different classes of lipids, including fatty acid, cholesterol, and sphingolipids. These peptides mainly target a conserved gp41 pocket site and show potent activity against HIV-1, HIV-2, and even SIV (Simian Immunodeficiency Virus) [273,274]. Other sets of peptides have been engineered with a similar approach on the C4 domain of gp120 to inhibit the interaction between the CD4 receptor and gp120 [277]. Peptide C4 (419–436) and, in particular, its head-to-tail polymeric form was found to block the binding of CD4 to gp120 [281]. Moreover, a peptide candidate for binding the site on the CD4 protein was identified by epitope mapping of a set of CD4-specific monoclonal antibodies using large CD4-derived synthetic peptides [276]. Through positional scanning libraries, overlapping peptide libraries, and similar approaches, several other peptides against HIV-1 have been identified and optimized [272,282].

Synthetic peptides have also attracted great attention in the treatment of influenza. In this case, antiviral chemotherapy makes use of two classes of drugs. The first includes inhibitors of the M2 proton-selective ion channel protein, such as amantadine and its derivative rimantadine [283,284]. The second are neuraminidase (NA) inhibitors such as oseltamivir and zanamivir [285]. Both are used against the influenza virus; however, drug-resistant strains have been already isolated [286,287,288] and new therapeutics are becoming a global health priority. In this context, a novel peptide inhibitor (errKPAQP) against influenza NA protein has been recently identified from a chemical peptide library made of L and D amino acids. One selected peptide binds NA with nanomolar affinity and inhibits infection of H1N1 in MDCK cells at μM concentrations. Additionally, treating infected mice with the peptide reduced lung tissue damage, viral inflammation, and mortality [289]. Other peptides were developed against the influenza A virus hemagglutinin (HA). HA is presented on the viral surface as a homotrimer containing disulphide-linked HA1 and HA2 subunits. The infection event is triggered through the binding of HA1 on the surface of the host cell expressing sialylated receptors followed by virus entry by endocytosis. In 2012, peptides derived from the C-lobe of the bovine lactoferrin (bLf), which significantly impairs the activity of HA, were proposed [290]. Starting from protein–protein docking simulations, a set of peptides mimicking the predicted interaction interfaces between bLf and HA proteins were identified and three peptides with significant antiviral activity were designed and tested [290]. Further studies on these peptides led to a set of tetrapeptides with broader anti-influenza activity, and one of these provided higher anti-influenza activity than the parental peptide in neutralization assays in MDCK cells [291]. Jones et al., tested a panel of cell-penetrating peptides for their anti-influenza activity [292]. They showed that a 20-mer peptide with entry-blocking activity denoted EB, derived from the fibroblast growth factor 4 (FGF-4) signal sequence, displayed a broad-spectrum antiviral activity against influenza viruses both in vitro and in vivo. Preventing attachment to host cells, the anti-viral peptide provided protective activity in mice at low micromolar concentrations likely interacting with the HA protein. A set of 12–16 residue-long anti-viral peptides termed FluPep (FP) was also developed against various influenza virus subtypes [293]. They are peptides derived from FP1, also known as Tkip peptide, designed based on the region of a suppressor of cytokine signaling (SOCS), which is active in modulating inflammatory cytokine responses in pox viruses [294]. Notably, one of the FP peptides (FP4: RRKKWLVFFVIFYFFR) showed nM efficacy against Influenza A virus subtypes H1, H3, and H5 in a plaque-reduction assay. Peptide inhibition of viral replication resulted from its interactions with HA and block of attachment to cells, or even reattachment and infection of neighboring cells. To compensate for the high hydrophobic features of FP peptides and to enhance their solubility, Alghrair and colleagues conjugated the FP peptide to gold and silver nanoparticle. The peptide-NP conjugates showed improved solubility and activity in plaque assays in MDCK cells [295]. Other peptides were also designed based on broadly neutralizing antibodies (bnAbs) targeting HA protein. A linear peptide named P1 (sequence SQLRSLEYFEWLSQ) was designed from the complementarity-determining region of the heavy chain and of the framework region of two bnAbs (called FI6v3) [296]. The P1 sequence was next optimized preparing and screening a new library of peptides containing several unnatural amino acids, leading to an 11-mer cyclic peptide named P7 (sequence Nva-Orn-meLEYchlFEWLS-βAla) that targeted Influenza A viruses (H1N1, H5N1) with a 100-fold improved potency compared to P1. P7 binds HA preventing its conformational rearrangement, and viral fusion with the host cell membrane [296]. Synthetic peptide libraries were also been used recently by Hejdanek and colleagues [297], to optimize a small peptide inhibiting the complex between PA and PB1 subunits of influenza virus RNA-dependent RNA polymerase (RdRp). Starting from the biologically active 14-mer fragment, called PB1-0 [298], capable of inhibiting the formation of complexes with an IC50 in the low nM range, they designed and synthesized a set of N- and C-terminally truncated peptides, shortening the active sequence to 10 residues. After further modification of the sequence a new optimized peptide (sequence DYNPYLLFLK) called PB1-11, was selected as capable of inhibiting the target interaction with an IC50 of 15 nM [297]. Noteworthy, the 3D structure of the complex between the shorter modified peptide and the C-terminal region of PA was determined, providing significant information for structure-based drug design. More recently, starting from PB1-11, another research group [299] further optimized the peptide by generating a set of mutated peptides and bi-cyclized peptides that are converted to linear peptides in the cells [300]. These modifications led to significant improvement of complex inhibition, increased transmembrane transport, increased early endosome escape and to antiviral activity in cellular assays.

Synthetic peptide libraries have also had a great impact on the fast development of peptide-based compounds against the severe acute respiratory syndrome (SARS) caused by SARS coronavirus. SARS-CoV-1 first appeared in China in 2002 [301]. The key step of viral infection of SARS is the interaction of the viral structural Spike-protein (S) and the host receptor angiotensin-converting enzyme 2 (ACE2) needed for cellular entry. Many research groups have strived to target S-ACE2 interaction of SARS-CoV-1. Several studies have led to the identification of different peptides with antiviral activity through the screening of sets of peptides designed on the S protein sequence [302,303]. In particular, Hu et al. [304] using a 10-mer overlapping peptide library, spanning the major structural proteins of SARS-CoV, identified a fragment encompassing residues S471–503 of protein S, located in the receptor binding domain (RBD) of SARS-CoV, able to specifically blocks the binding between RBD and ACE2. The peptides that inhibited SARS-CoV entry into host cells in vitro and are now in preclinical trials. Severe acute respiratory syndrome coronavirus 2 (SARS-CoV-2) is a strain of coronavirus that causes COVID-19 (coronavirus disease 2019), the severe respiratory disorder responsible for a still ongoing pandemic. Since no antiviral and/or FDA-approved vaccines were available to combat the disease at the time of the pandemic onset the scientific community made huge efforts to develop new therapeutics. As for SARS-CoV1, in the initial step, the receptor-binding domain (RBD) of the S protein interacts with the host ACE2 receptor. To try to block this PPI, a significant number of peptide molecules based both on the ACE2 and the RBD domain of the Spike have been studied [305]. Additionally, peptides against proteases have been developed using combinatorial chemistry approaches. The PLpro and Mpro enzymes of SARS-CoV-2 have been also targeted by peptide-based inhibitors. Using a combinatorial library, one group has developed peptide inhibitors against these proteases [306]. First, substrates for both proteases were identified using a library containing proteinogenic and non-proteinogenic amino acids. Then, the best substrate candidates were converted into peptide inhibitors adding an irreversible reactive group at the C-terminus. Mpro inhibitor QS1 (Ac-Abu-Tle-L-Q-VS), and PLpro inhibitors VIR251 and VIR250 (Ac-hTyr-Dap-G-G-VME and Ac-Abu(Bth)-Dap-G-G-VME) have been identified in this way. Elucidation of the crystal structure of S protein RBD in complex with ACE2 (pdb codes: 6MOJ and 6M17), has clarified the hotspots of this interaction at the atomic level facilitating the development of inhibitors. A peptide of 23-residue (SBP1, IEEQAKTFLDKFNHEAEDLFYQS), derived from the α1 helix region of human ACE2 [305], and a peptide named EK1 (SLDQINVTFLDLEYEMKKLEEAIKKLEESYIDLKEL), derived from the heptad repeat 2 (HR2) domain of the S protein [307], have been reported as successful inhibitors. They can disrupt the PPI, binding strongly to the mutual recognition sites on the targets [307]. Even if these peptides show strong antiviral activity in vitro, they did not exhibit the expected results in vivo, suggesting that further modifications of the linear peptides are needed. Significant advances in the design of peptide-based compounds against RBD-ACE2 interaction have been also made using peptide or peptide-based compounds mimicking discontinuous binding sites. RBD interacts with the ACE2 receptor through h1 and h2 α-helices, and triggers the fusion of the cell and viral membranes to permit the viral genome to penetrate the cytoplasm [308]. Sadremomtaz et al. in 2022 [309] explored the protein contact surface and used molecular dynamics simulations to locate the main hotspots on α1, α2, α3, β3, and β4 of ACE2. Based on combinations of the various hotspots, they prepared and tested a focused library of discontinuous peptides (six compounds), some of which demonstrated high efficacy (∼10 nM as determined by microscale thermophoresis) in binding soluble RBD (S-RBD) and antagonizing the SARS-CoV-2 S-RBD:ACE2 interaction in bioluminescence-based assay. However, the linear peptides identified did not exhibit antiviral activity in cells, as also found with previously reported peptide antagonists of S-RBD:ACE2 [310,311]. These findings suggested that further optimizations of peptides, such as lipidation, were needed. Along these lines, Sidorova et al. designed and synthesized several peptides derived from α-helices h1 and h2 of ACE2 linked by disulphide bridges at different positions to generate chimeric molecules. Data showed that the dimeric peptides have an improved affinity for SARS-CoV-2 RBD, likely due to their higher degree of helicity than monomeric precursors [312]. Further stabilization of the peptide structure and optimization of the sequence and solvent exposure is likely required to obtain molecules able to properly interact with the receptor in vivo. Still searching for peptide inhibitors capable of eradicating COVID-19 infections, Weissenborn et al. (2022) [313] prepared a series of conformationally constrained peptide variants of the computationally designed miniprotein LCB1 using the 3D structure of its complex with RBD (pdb code: 7JZU). Surprisingly, some peptides retained the same ability of LCB1 to inhibit the interaction of RBD with ACE2, as well as to neutralize SARS-CoV-2 activity. Furthermore, a cyclic peptide with higher activity and proteolytic stability was designed, identifying a potential candidate for SARS-CoV-2 therapy. Using overlapping polypeptides, alanine mutants, and single synthetic peptides, Zhou et al. in 2020 identified two antigenic regions of the P30 immunogenic protein of African swine fever virus (ASFV), which is responsible a lethal hemorrhage and highly contagious diseases in swine [314]. As no ASF vaccine is currently available, highly specific and sensitive early diagnosis is crucial for the immediate detection of infected herds and disease observation. The results of this study have thus raised interest in the valuable insight provided into the antigenic regions of ASFV P30, which are critical to lay the basis for ASF vaccine research and serological diagnosis. In a study on the bacterium Rhodococcus equi, an important pulmonary pathogen in foals, Vanniasinkam et al. (2018) designed a set of overlapping peptides based upon a known amino acid sequence of the bacterium target protein VapA (Virulence-associated Protein A). The fragments synthesized as biotinylated peptides and screened by ELISA lead to the identification of a linear B-cell epitope, corresponding to a 20-mer long peptide of the VapA protein [172].

The interaction between the p6 domain of the HIV Gag protein and the human UEV domain TSG101 protein drives HIV budding. Lennard et al. in 2019 screened an alanine scanning library to identify a biologically active and permeable peptide-based compound of these PPIs starting from a known p6/UEV inhibitor peptide. Amino acids crucial for the activity of the parent peptide were identified using alanine scan arrays and a series of unnatural peptide analogs. The most powerful molecule prevented the p6/UEV interaction with an IC50 of 6.17 ± 0.24 μM and bound to UEV with a KD of 11.9 ± 2.8 μM. This compound resulted cellular permeable and active in a cellular virus particle budding test with an IC50 of ~2 μM [315].

Recent studies have demonstrated that COVID-19-recovered patients have an increased risk of contracting T2D or diseases correlated with amyloid [316,317]. Nyström et al. [318] used a 316 synthetic overlapping peptide library generated from a peptide scan through the entire SARS-CoV-2 Spike protein, using TEM discovered regions responsible for amyloid-like fibril formation. Interestingly, fibril formation occurred when SARS-CoV-2 Spike protein was co-incubated with the protease neutrophil elastase (NE) which is overexpressed at inflamed sites of viral infection and efficiently cleaves the Spike protein. In vitro, fragment 194–203 of SARS-CoV-2 Spike protein forms fibrils with NE. As it is known that Spike protein affects the formation of persistent amyloid-like microclots in human blood [319], COVID-19 could cause other potential pathologic diseases involving amyloidogenic processes in vivo in the long period.

## 7. Conclusions and Perspectives

The literature survey here reported illustrates the important role that peptide-based combinatorial libraries are playing in the discovery of PPI modulating compounds, an emerging class of therapeutic agents that are assuming an increasingly crucial role in the panorama of drugs that will underpin personalized medicine. Their growing importance is mainly due to the continuous discovery of new molecular mechanisms of diseases that specifically involve altered or improper interactions between proteins that are perhaps mutated, undergo structure alterations or are overexpressed in cellular compartments where they do not normally reside.

Inhibitors of PPIs also have a decisive role in basic science as they provide insightful information on the molecular mechanisms underlying intricate biological processes. The main advantages on the use of peptides in this specific field and more in general in drug discovery is the broad chemical diversity they offer thanks to the variety of building blocks usable for their assembling and the different structures they can adopt, thus ensuring high affinity and selectivity which translate into reduced undesired interactions and side effects in in vivo contexts. Additionally, given the generally ample and multifunctional surfaces and high flexibility, peptides are particularly suitable to target the large interfaces that underlie protein–protein contacts.

The design and development of peptides able to modulate physio-pathological processes are typically based on distinct but increasingly complementary methodologies that are generally grouped in either rational or combinatorial approaches. Choosing the one or the other or even integrating the two is mostly dictated by the availability of robust structural data on the target protein complex. Indeed, rational design approaches can only be pursued by having sufficiently detailed structural information, in the absence of which combinatorial approaches become a valid option for identifying bioactive peptides. The use of combinatorial libraries involves the preparation of large sets of synthetic peptides (libraries) and their screenings, allowing for the selection of compounds with a specific activity. The rational design and combinatorial approaches are not mutually exclusive and are often used in combination applying combinatorial methodologies after an initial phase of rational and/or computational design, which can help circumscribe the diversity and molecular space to be explored with combinatorial libraries, thus reducing both synthetic and screening efforts and further eliminating fewer specific ligands [21,24,25].

In this perspective, the library screening approach in the drug discovery field has been significantly re-modulated over the years. Indeed, while initial applications were more specifically focused on increasing the number and diversity of compounds, more recently, molecular design has gained an progressively important role leading to mixed approaches where smaller but target-focused libraries are designed ad hoc to maximize the likelihood of being active in biological assays or even to improve the drug-like properties, pre-incorporating structural features that also ameliorate the final ADMET profiles.

The recent literature shows that combinatorial libraries are particularly powerful and versatile to develop peptide-based PPI inhibitors, since, if properly managed in terms of initial design of structures, diversity, multiplicity and biological assays can rapidly lead to new hit or drug candidates. They offer the great advantage of increased diversity deriving from the introduction of non-natural building blocks and non-canonical scaffolds leading to cyclic, multicyclic, side-chain decorated and branched structures, beyond the possibility to more easily control the number of different components by varying the synthetic steps and the number of building blocks. Nevertheless, biological libraries are also very effective and the recent introduction of post-translational chemical modifications in phage display libraries is opening up a new unexplored field where chemistry and biology are combined and integrated.

Considering the impressive results achieved in the last two years using machine learning approaches in the accurate prediction of protein three-dimensional structures, for example, AlphaFold [55,56,57,58,59], peptide-based chemical combinatorial approaches are expected to soon have a major role in the development of effective molecule able to modulate PPI. Indeed, combinatorial chemistry may represent a valuable tool to validate the putative functional protein–protein interfaces that these predictive approaches can generate even at interactome level. Moreover, the inability of these predictive approaches of estimating the effects in protein folding and/or in protein–protein interaction of minimal amino-acid substitutions renders combinatorial chemistry an important tool for optimizing PPI modulators.
